# Non-viral Delivery of Nucleic Acids: Insight Into Mechanisms of Overcoming Intracellular Barriers

**DOI:** 10.3389/fphar.2018.00971

**Published:** 2018-08-21

**Authors:** Mikhail Durymanov, Joshua Reineke

**Affiliations:** Department of Pharmaceutical Sciences, College of Pharmacy and Allied Health Professions, South Dakota State University, Brookings, SD, United States

**Keywords:** polyplexes, lipoplexes, gene delivery, siRNA delivery, intracellular trafficking, endosomal escape

## Abstract

Delivery of genes, including plasmid DNAs, short interfering RNAs (siRNAs), and messenger RNAs (mRNAs), using artificial non-viral nanotherapeutics is a promising approach in cancer gene therapy. However, multiple physiological barriers upon systemic administration remain a key challenge in clinical translation of anti-cancer gene therapeutics. Besides extracellular barriers including sequestration of gene delivery nanoparticles from the bloodstream by resident organ-specific macrophages, and their poor extravasation and tissue penetration in tumors, overcoming intracellular barriers is also necessary for successful delivery of nucleic acids. Whereas for RNA delivery the endosomal barrier holds a key importance, transfer of DNA cargo additionally requires translocation into the nucleus. Better understanding of crossing membrane barriers by nucleic acid nanoformulations is essential to the improvement of current non-viral carriers. This review aims to summarize relevant literature on intracellular trafficking of non-viral nanoparticles and determine key factors toward surmounting intracellular barriers. Moreover, recent data allowed us to propose new interpretations of current hypotheses of endosomal escape mechanisms of nucleic acid nanoformulations.

## Introduction

Cancer gene therapy remains a significant challenge due to numerous barriers limiting delivery of genetic cargo. In contrast to a vast majority of nanoformulated chemotherapeutic drugs, nanoparticles for nucleic acid delivery have to reach the specific intracellular compartment; either cytosol for siRNA and mRNA, or nucleus in case of DNA. The importance of overcoming these generally conserved (applicable to numerous cell types within the cancer milieu) intracellular barriers is increasing as additional genetic manipulation technologies, such as the CRISPR/Cas9 system, will all require delivery to specific intracellular compartments to be effective and clinically relevant.

Despite advantages of viral vectors in terms of gene delivery efficacy, their use may cause immune responses and severe side effects (Raper et al., 2003; Manno et al., 2006; Howe et al., 2008) resulting in limited and very cautious clinical use. In this context, synthetic carriers able to form complexes with nucleic acids, and protect them from extra- and intracellular nucleases, are considered an alternative to viral vectors. Viral particles possess innate machinery to overcome cellular barriers, however, engineering of non-viral carriers requires great effort to rationally design nucleic acid nanoformulations to overcome the same barriers. Development of cationic polymers and lipids with their ability to deliver genetic material into cells gave rise to extensive studies of the mechanisms underlying transfection properties of these carriers. Obviously, this knowledge would provide the basis for future improvement of their efficacy to the level comparable with viral counterparts. To date, the progress in this direction is still insufficient.

There are numerous reviews on the topic focusing mostly on recent advances in chemistry of nanocarriers for improving nucleic acid delivery without detailed description of the mechanisms (Yao et al., 2013; Ma, 2014; Hill et al., 2016; Stewart et al., 2016a,b; Lai and Wong, 2018). The purpose of this review is to discuss intracellular barriers for non-viral delivery of nucleic acids, their significance, and mechanisms, which are exploited by different types of artificial gene carriers to overcome the key barriers. We also summarized here recent advances on how intracellular delivery of nucleic acid nanoformulations can be improved. Special attention is given to the endosomal barrier, particularly in light of recent findings obtained by time-lapse microscopy of living cells. Parallel consideration of these data and the endosome maturation process allowed us to propose our interpretation of endosomal escape mechanisms for nucleic acid nanoformulations.

## Intracellular Trafficking of Nucleic Acid Nanoformulations in Cancer Cells

**FIGURE 1:**
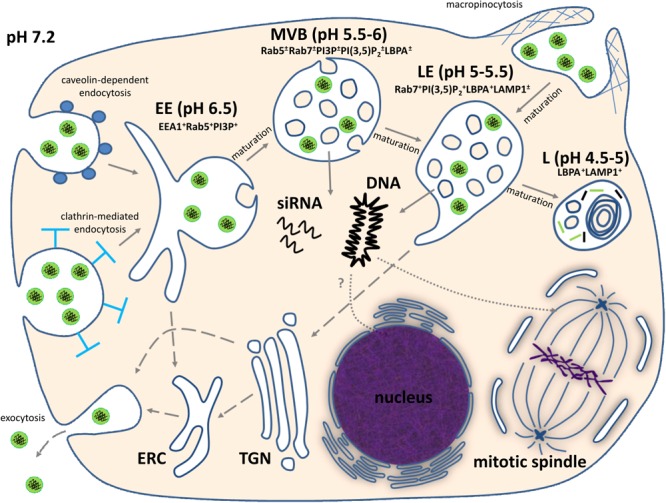
Intracellular trafficking of non-viral nucleic acid nanoformulations. Comments can be found in the text. EE, early endosome; MVB, multivesicular body; LE, late endosome; L, lysosome; ERC, endocytic recycling compartment; TGN, *trans*-Golgi network; EEA1, early endosome antigen 1; PI3P, phosphatidylinositol 3-phosphate; PI(3,5)P_2_, phosphatidylinositol (3,5)-*bis*phosphate; LBPA, lyso*bis*phosphatidic acid; LAMP1, lysosomal-associated membrane protein 1. “+” and “±” mean high and moderate levels of abundance, respectively.

Similar to some types of viral vectors, artificial nanoparticles for nucleic acid delivery penetrate the cells exploiting endocytic mechanisms. Nanoparticles can enter cancer cells via different types of endocytosis and/or macropinocytosis (**Figure 1**). Internalization through phagocytic pathways occurs primarily in monocytes/macrophages, neutrophils, and dendritic cells (Sahay et al., 2010), and usually is not attributed to cancer cells.

Upon uptake, endosomal vesicles undergo a maturation process driven mainly by phosphatidylinositols (PIs) in the cytosolic leaflet of the vesicle bilayer (Bohdanowicz and Grinstein, 2013) and Rab GTPases (Hutagalung and Novick, 2011). During maturation, biochemical characteristics and morphology of vesicles significantly change (**Figure 1**). In particular, early endosomes lose tubular membrane structures which become the endocytic recycling compartment and provide retrograde transport of phospholipids and receptors to plasma membrane. Some fraction of internalized nanoparticles with genetic payload might be recycled by this route (Gonçalves et al., 2004; Sahay et al., 2013), but this process seems to depend on physicochemical properties of nanoparticles and cancer cell type. The membranes of early endosomal compartments are enriched with phosphatidylinositol 3-phosphate (PI3P), which provides interaction of the vesicle with cytosolic early endosome antigen 1 (EEA1), an endosomal sorting complex required for transport (ESCRT) machinery, and Rab5 GTPase (Bohdanowicz and Grinstein, 2013). All these proteins are required for sorting and progression of early endosomal vesicles to degradative compartments. It should be noted, that internalized nucleic acid nanoformulations are sorted to this degradative pathway. Maturation of early endosomes leads to their transformation into multivesicular bodies (MVBs) containing intraluminal vesicles (ILVs). This process is mainly governed by gradual replacement of Rab5 with Rab7, which regulates endo-lysosomal morphogenesis (Hutagalung and Novick, 2011). MVBs are characterized by an acidic luminal pH promoted by V-type H^+^-ATPase. Moreover, MVBs gradually acquire *bis*(monoacylglycero)phosphate (BMP), also known as lyso*bis*phosphatidic acid (LBPA), which along with ESCRT-III complex is involved into ILV formation (Williams and Urbé, 2007; Bissig and Gruenberg, 2014). Further conversion of MVBs to late endosomes leads to elevation of Rab7 and BMP/LBPA content, and increase of ILV number due to inward budding of the MVB limiting membrane. Additionally, the limiting membranes of late endosomal compartment contain a high level of phosphatidylinositol (3,5)-*bis*phosphate [PI(3,5)P_2_], which regulates sorting of endocytic membranes for delivery to the *trans*-Golgi network (Bohdanowicz and Grinstein, 2013). This mechanism of *trans*-Golgi network-mediated transport also may contribute to partial recycling of nucleic acid nanoformulations to the extracellular milieu (Sahay et al., 2013). Transformation of late endosomes into lysosomes is accompanied by acquisition of lysosomal-associated membrane proteins (LAMPs), formation of multilaminar structures enriched with BMP/LBPA (Möbius et al., 2003), highly acidic pH level, and activation of lysosomal hydrolases which can destroy genetic payloads of therapeutic nanoparticles. For successful delivery of nucleic acids, overcoming endosomal barrier is mandatory and preferable before conversion of endosomes into lysosomes with activated hydrolases (**Figure 1**).

For delivery of DNA another essential barrier is the passage through the nuclear envelope. Nuclear entry of macromolecules occurs via channels of the nuclear pore complex (NPC) in a size-dependent manner. For example, macromolecules with size less than 9 nm (or 40 kDa molecular weight) are able to diffuse passively through the NPC. Import of larger molecules can occur through an energy- and signal- dependent active process (Talcott and Moore, 1999; Gamini et al., 2014). Regarding DNA transfer, microinjection experiments have shown that 200–300 bp is the maximal length of linear DNA that can cross the NPC relatively freely (Ludtke et al., 1999). Since the average length of therapeutic gene expression cassettes are a few kbp, their NPC-mediated transfer into the nucleus is improbable. However, a nuclear envelope temporarily breaks down during mitosis and provides opportunity for DNA translocation to the nuclei of daughter cells, although non-identified mitosis-independent mechanism also can be involved into DNA uptake by the nucleus (**Figure 1**).

Thus, delivery of genetic cargo to cytosol or nucleus is a complicated multi-step process affected by numerous factors. The success of nucleic acid delivery is a result of overcoming all mentioned barriers for which mechanisms and impacts are discussed below.

## Internalization of Nanoparticles With Genetic Payload

Endocytic routes are the primary internalization mechanism for nucleic acid nanoformulations. Although non-endocytic mechanisms such as fusion of lipoplexes with the plasma membrane and pore formation on the cell membrane have been reported, the data confirming their involvement in nucleic acid delivery process are controversial, and direct proofs are still lacking (Xiang et al., 2012). Since endocytic pathways of non-viral carriers have been thoroughly analyzed in numerous reviews (Xiang et al., 2012; El-Sayed and Harashima, 2013; Perez Ruiz de Garibay, 2016), here, we will focus only on a few aspects of endocytosis which affect transfection efficacy.

To date, only four endocytic pathways of non-viral nanoparticle uptake have been determined for cancer cells including clathrin-dependent, caveolae-dependent, flotillin-mediated, and macropinocytosis (Perez Ruiz de Garibay, 2016). The determination of endocytic route depends on multiple factors including both cell line and nanocarrier parameters such as chemical nature, presence of ligand moiety, and nanoparticle size and surface charge (Xiang et al., 2012). It is generally accepted that positive charge promotes nanoparticle interaction with anionic glycosaminoglycans on the cell surface and enhances their uptake (Kopatz et al., 2004). Of importance to emphasize is that all endocytic pathways result in the endolysosomal system pathway. It has been hypothesized earlier that targeting caveolae-mediated pathway can avoid a lysosomal destination for nucleic acid nanoformulations (Rejman et al., 2005). However, later study of this type of endocytosis confirmed lysosomal transport of the internalized cargo (Engel et al., 2011). Despite similarity in the trafficking pathway, the specific endocytic pathway affects kinetics of non-viral nanocarrier internalization and total uptake potential. Some studies for both lipoplex (Sakaguchi et al., 2008) and polyplex (Trusov et al., 2011; Ulasov et al., 2011) nanoparticles demonstrated positive correlation of internalization rate and transfection efficacy. Additionally, importance of increasing uptake as a design criteria for nanocarriers was indicated by Bishop et al. The authors found that an insignificant amount of poly(beta-amino ester)-based polyplexes was internalized by human glioblastoma cells in 2D culture (Bishop et al., 2016). In this connection, modification of RNA- or DNA- containing nanoparticles with a ligand moiety seems to be beneficial because it facilitates binding of these nanoformulations with the cell surface and induces their endocytosis in a complex with rapidly internalizing receptors. As a result, improved transfection efficacies of cancer cells have been observed for nanovehicles containing a ligand to transferrin (Sakaguchi et al., 2008; Zhang et al., 2015), α_ν_β_3_ integrin (Ng et al., 2009; Mohammed-Saeid et al., 2017), melanocortin-1 (Durymanov et al., 2012), EGF (Kloeckner et al., 2006), and folate (Liu et al., 2012; Jones et al., 2016) receptors as compared with non-targeted counterparts. Additionally, it cannot be excluded that nanoparticle internalization in a complex with some of the mentioned receptors might decrease the rate of the recycling process via the exocytosis pathway, which was reported for non-targeted lipid nanoparticles (Sahay et al., 2013) and polyplexes (Gonçalves et al., 2004).

## Endosomal Escape

### Lipoplex-Formulated Release of Genetic Payload From Endosomes

**FIGURE 2:**
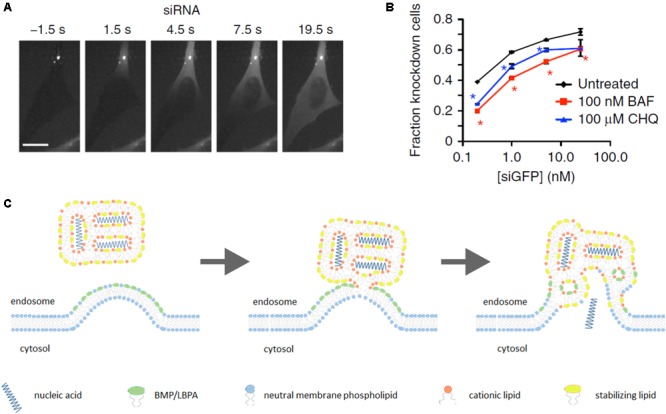
Lipoplex endosomal escape. Escape of lipoplex-formulated genetic cargo from an endosome in HeLa cells takes seconds followed by diffusion of siRNA throughout entire cytosol **(A)**. Efficacy of GFP knockdown in HeLa-GFP cells upon incubation with lipoplexes carrying siGFP in the presence of inhibitors of endosomal acidification bafilomycin A1 (BAF) or chloroquine (CHQ) **(B)**. “Lipid-mixing” mechanism of endosomal escape of lipoplex-formulated nucleic acids in assumption of primary role of BMP/LBPA, concentrated in the luminal leaflet of endosomal membrane in area of ILV formation (see the text for additional comments) **(C)**. **(A,B)** Figures are reprinted by permission from Nature Publishing Group (Wittrup et al., 2015).

In recent years, a substantial breakthrough in elucidation of endosomal escape of nucleic acid nanoformulations has been achieved due to the use of spinning-disk microscopy for observation of nanoparticle intracellular trafficking in real time. Exploiting this technique has shown that Lipofectamine 2000-based lipoplexes release their genetic payload (siRNA) starting after 5–15 min of uptake by HeLa cells. Endosomal release of siRNA occurs instantly, and is followed by rapid (10–20 s) diffusion of siRNA throughout entire cytosol (**Figure 2A**). At the same time, a significant fraction of cargo stayed within endosomes as well as the lipid part of lipoplex (ur Rehman et al., 2013; Wittrup et al., 2015). Thus, genetic payload is released uncoated. It was also found that endosomal escape takes place in vesicles with EEA1^-^Rab5^+^Rab7^+^Rab9^±^LAMP1^-^ phenotype that corresponds to maturing but not late endosomes. Further, inhibition of endosome acidification with bafilomycin A impaired efficacy of gene silencing by 10–40% depending on siRNA dose (**Figure 2B**) (Wittrup et al., 2015).

These findings generally do not contradict an earlier proposed explanation of lipoplex endosomal escape, also known as “lipid-mixing” mechanism. This hypothesis was suggested in 1996 basing on *in vitro* mimicking of lipid/DNA nanoparticle interactions with membranes containing the negatively charged phospholipid phosphatidylserine (PS) (Xu and Szoka, 1996). According to this hypothesis, lipid/DNA (or lipid/RNA) complexes destabilize endosomal membrane due to flip–flop translocation of anionic lipids (mainly PS) from cytosolic leaflet to luminal side. Following this, these negatively charged phospholipids electrostatically interact with cationic lipids in lipoplexes resulting in displacement of genetic cargo. This then leads to production of non-bilayer structures (inverted hexagonal H_jj_ phase) in endosomes and release of nucleic acids to the cytosol (Xu and Szoka, 1996; Hafez et al., 2001).

However, some questions still remain. For example, why lipoplexes discharge genetic payload in maturing endosomes, whereas the PS content in these compartments is as a minimum twofold less than in early endosomes (Kobayashi et al., 1998)? Why a pH-dependence of endosomal escape has been observed? Probably, more detailed consideration of endosome maturation process may help to find possible explanations of these facts. It should be noted that besides PS, there are other negatively charged lipids in the endosomal bilayer such as phosphatidylinositols PI3P and PI(3,5)P_2_. However, they are also localized in the cytosolic leaflet of endosomal bilayers with PS, and their abundance is relatively low. An additional consideration is that MVBs are enriched with BMP/LBPA, an unconventional anionic phospholipid, which may play a primary role in endosomal release of genetic payload from lipoplexes.

To determine localization of BMP/LBPA in MVBs, some studies with monoclonal antibody 6C4 against this phospholipid were carried out. Microinjection of this antibody into cells did not lead to MVB/lysosome targeting, but it was obtained after staining with prior fixation and permeabilization. Additionally, fluorescence correlation spectroscopy demonstrated that 6C4 only binds sonicated, but not intact late endosomes (Kobayashi et al., 2001). Therefore, obtained results revealed the lack of BMP/LBPA in cytosolic leaflet. Furthermore, anti-BMP/LBPA antibodies demonstrated high co-localization extent with membranes of ILVs, and only minor presence in limiting MVB membrane at sites of intraluminal invaginations (Kobayashi et al., 1998, 2001; Möbius et al., 2003). It means that cationic lipids of lipoplex can directly interact with BMP/LBPA-rich areas on the luminal side of limiting MVB and ILV membranes. It is thought that in mammalian cells BMP/LBPA may be involved in ILV formation along with ESCRT due to inward budding of the limiting MVB membrane (Falguières et al., 2009). BMP/LBPA is an inverted cone-shaped phospholipid (Williams and Urbé, 2007) promoting positive membrane curvature (Harayama and Riezman, 2018). It has been shown *in vitro* that BMP/LBPA-containing liposomes are able to form ILVs at acidic intraluminal pH (pH-dependent) resembling MVBs (Matsuo et al., 2004). Perhaps, acidification of endosomal lumen causes partial protonation of phosphate groups of BMP/LBPA that minimizes electrostatic repulsion of anionic headgroups and alleviates clustering of BMP/LBPA favoring inward curvature of the limiting membrane toward endosome lumen. Inhibition of endosomal acidification might impede BMP/LBPA clustering and interaction with positively charged lipoplexes decreasing efficacy of endosomal escape.

Thus, BMP/LBPA is a stronger candidate for induction of nucleic acid endosomal escape than PS because it can directly interact with positively charged lipoplexes at areas of MVB limiting membrane invaginations (**Figure 2C**), whereas PS flipping from cytosolic leaflet is thermodynamically restricted because of charged headgroups (Sprong et al., 2001). Furthermore, PS has a cylindrical shape, favoring bilayer formation (Sprong et al., 2001), whereas BMP/LBPA is a wedge-shaped phospholipid which destabilizes the bilayer phase upon interaction with cationic cone-shaped lipids. As was mentioned above, the phenotype of endosomes where release of genetic cargo occurs coincides with MVBs and start of BMP/LBPA generation. Finally, our hypothesis about the primary role of BMP/LBPA in endosomal release may explain why inhibition of pH affects siRNA-mediated knockdown efficacy.

### Overcoming Endosomal Barrier by Polyplexes

Intravital microscopy enabled valuable information to be obtained about intracellular trafficking and endosomal release of polyplex-formulated siRNA and plasmid DNA. It was found that polyethylenimine (PEI)-based polyplexes escape from endosomes at approximately 30 min after uptake by HeLa cells. This process occurs instantly and is accompanied by partial release of PEI together with genetic payload. Discharge of genetic payload in case of polyplexes occurs from LAMP-1-positive vesicles corresponding to late endosomes/lysosomes (ur Rehman et al., 2013). Furthermore, endosomal acidification plays a crucial role in endosomal escape because both genetic cargo release and transfection efficacy were virtually inhibited by bafilomycin A (Kichler et al., 2001; ur Rehman et al., 2013).

**FIGURE 3:**
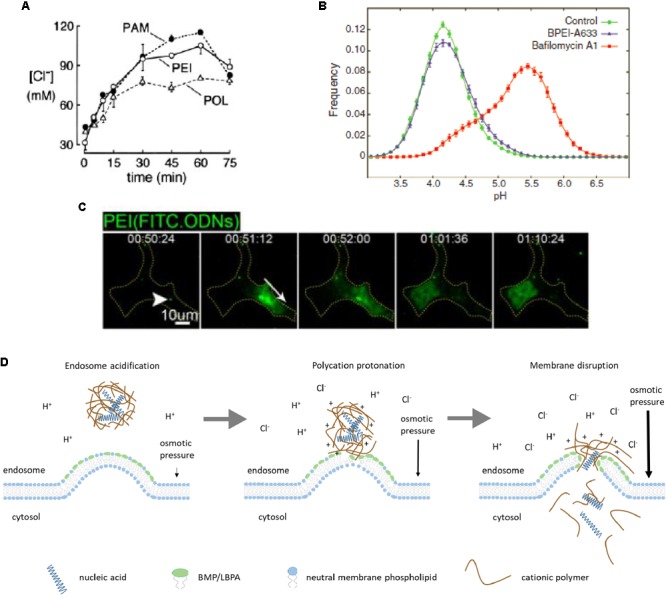
Polyplex endosomal escape. Accumulation of chloride in endosomal vesicles upon cellular uptake of polyamidoamine (PAM), polyethylenimine (PEI), and polylysine (POL) reflects higher buffering capacity of PAM and PEI **(A)**. Lack of acidic endosomal pH increase in vesicles containing branched PEI (BPEI), compared to bafilomycin A-treated cells, contradicts the “proton sponge” hypothesis **(B)**. Released polyplex-formulated genetic cargo (FITC-labeled oligodeoxyribonucleotides, ODNs) from an endosome in HeLa cells spreads in a single direction (white arrow) **(C)**. Proposed mechanism of endosomal escape of polyplex-formulated nucleic acids driven by osmotic pressure and local permeabilization of endosomal membrane due to electrostatic interactions between polycation and anionic phospholipids like BMP/LBPA **(D)**. Figure adapted with permission from: **(A)** (Sonawane et al., 2003), ASBMB; **(B)** (Benjaminsen et al., 2013), Elsevier; **(C)** (ur Rehman et al., 2013), copyright (2013) American Chemical Society.

Interestingly, endosome acidification plays a key role in the “proton sponge” hypothesis mechanism of polyplex endosomal escape proposed over 20 years ago (Boussif et al., 1995). This hypothesis postulates that the buffering capacity of polyamine carriers leads to osmotic rupture of endosomal membrane and release of polyplexes into cytosol. Experiments with PEI-treated cells demonstrated increased accumulation of endosomal chloride (**Figure 3A**) responsible for osmotic swelling of endosomes, followed by the lysis of endocytic PEI-containing vesicles that supported the “proton sponge” hypothesis (Sonawane et al., 2003). However, a more recent study detected no change in endosomal pH within vesicles containing PEI (**Figure 3B**), whereas according to the “proton sponge” mechanism, pH should increase due to PEI buffering capacity (Benjaminsen et al., 2013). This contradiction strongly challenged the “proton sponge” hypothesis. At the same time, different polyamines including poly-L-lysine, poly(amidoamine) and PEI can permeabilize supported lipid bilayers and even cell plasma membranes (Hong et al., 2006) that may also explain endosomal escape. Experimental data with bafilomycin A confirmed the importance of endosome acidification as mentioned above. It was also found that endosomal escape does not lead to complete endosome rupture as the “proton sponge” hypothesis postulates. Additionally, release of polyplex-formulated fluorescently labeled siRNA from endosomes spreads in a single direction (**Figure 3C**) (ur Rehman et al., 2013).

Our interpretation of these experimental data considers direct interaction of cationic part of polyplexes with negatively charged phospholipids, primarily with BMP/LBPA. Regarding buffering properties of the carriers, they could significantly facilitate endosomal escape. First, protonation of polycation may lead to more strong electrostatic interaction with negatively charged lipids, resulting in membrane permeabilization. Second, even though luminal pH does not increase, influx of chloride ions promotes osmotic swelling of endosomes. Upon reaching a critical value, osmotic pressure imparts mechanical impulse to nucleic acids and part of associated polycation leading to their discharge from endosome (**Figure 3D**). According to our model, both buffering capacity of polycation and its ability to interact with anionic lipids like BMP/LBPA and permeabilize endosomal membrane are important for endosomal escape.

### Crossing Endosomal Barrier by Inorganic Nanoparticles

Besides lipid- and polycation-based delivery systems, there are several types of inorganic materials which are exploited for nucleic acid delivery to tumors. Among them, calcium phosphate (CaP) composite is the oldest non-viral gene carrier; introduced in 1973 (Graham and van der Eb, 1973). CaP is a biocompatible and degradable material able to form a complex with nucleic acids and successfully deliver them to cells (Yao et al., 2013). Despite many years of use of this transfection agent, the mechanisms of overcoming the endosomal barrier remain elusive. In a study by Li et al. (2010), it was proposed that endosomal release of nucleic acids delivered by CaP is an osmotically driven process. Low luminal pH causes rapid dissolving of CaP that results in an increase of osmotic pressure and rupture of endosomal vesicles. One of the most important limitations of CaP precipitates is control of their size because CaP crystals grow over time (Ramamoorth and Narvekar, 2015). However, PEG conjugation or lipid coating approaches improved colloidal stability of these nanocarriers and led to clinically relevant *in vivo* efficacies (Ma, 2014; Pittella et al., 2014). Probably, increased interest to these delivery vehicles will result in more efforts to study endosomal nucleic acid release mechanisms.

Gold nanoparticles are promising inorganic materials for gene-based therapy of cancer. Their physical properties make it possible to cause plasmon resonance after near infrared (NIR) irradiation and control nucleic acid release *via* thermal and non-thermal mechanisms (Huschka et al., 2011, 2012). Along with controlled release of genetic payloads, NIR irradiation also causes endosomal disruption. At least two possible mechanisms are reported to be involved in endosomal escape depending on the mode of NIR irradiation. For both pulsed and continuous wave irradiation of high laser intensity, a thermal mechanism plays the major role (Carregal-Romero et al., 2012). Local heating of the surrounding area induces enhanced mobility and oscillations of endosomal phospholipids resulting in high membrane leakiness (Stewart et al., 2016b). However, endosomal escape of gold nanoparticles can be induced by low intensities of NIR irradiation. Authors also detected enhanced photochemical generation of reactive oxygen species (Krpetic et al., 2010), which can be an underlying mechanism of endosomal vesicles disruption resulting in release of nanoparticles into the cytosol. Thus, light-induced endosomal escape of gold nanoparticles potentially may improve selectivity of nucleic acid delivery to localized tumors.

Carbon nanotubes have a unique property of endocytosis-independent cell penetration due to the so-called “needle” mechanism and may completely avoid the endosomal barrier (Pantarotto et al., 2004). Thus, they are a promising carrier for nucleic acids transfer, though this gives rise to a potential issue of off-target delivery. However, several recent studies have challenged endocytosis-independent cellular internalization of carbon nanotubes (Kam et al., 2006; Becker et al., 2007), though the uptake pathway seems to depend on the length of nanotubes (Caoduro et al., 2017). Regardless, additional strategies have been proposed to improve nucleic acid delivery with carbon nanotubes (Li et al., 2015).

Two other types of inorganic delivery vehicles, namely, silica-based (Niut et al., 2012) and superparamagnetic iron oxide (Taratula et al., 2011; Luo et al., 2017) nanoparticles have been proposed as nucleic acid carriers due to their enhanced loading capacity and the possibility to monitor biodistribution, respectively. These materials cannot innately promote endosomal disruption and need additional chemical modifications to tune their endosomal escape capabilities.

### Strategies to Improve Endosomal Escape

It should be noted that there are numerous additional endosomal escape approaches for nucleic acid nanoformulations (Stewart et al., 2016a) (**Table 1**). First, co-treatment of cells with lysosomotropic agents such as chloroquine, sucrose, polyvinylpyrrolidone (PVP) and others can be used (Ciftci and Levy, 2001; Cheng et al., 2006). These agents accumulate in endosomes together with gene delivery nanoparticles, cause pH buffering and induce osmotic swelling of endosomal vesicles, minimizing damage of nucleic acid cargo and promoting more efficient release into cytosol. Chloroquine is the most widely used lysosomotropic agent. However, in spite of its relatively high *in vitro* efficacy and successful attempt of use to promote polyplex-mediated gene delivery to liver (Zhang et al., 2003), *in vivo* translation of this approach is limited because of high systemic toxicity (Cann and Verhulst, 1961). On the other hand, co-encapsulation of chloroquine with DNA or RNA in a single particle may overcome the need for systemic treatment. Bhattarai et al. (2010) fabricated mesoporous silica nanoparticles conjugated with PEG and poly(2-(dimethylamino)ethylmethacrylate) (PDEAEMA) for delivery of DNA or siRNA. Loading of these particles with chloroquine significantly improved efficacy of gene delivery or gene silencing in B16F10 melanoma cells. However, this strategy still has not been tested *in vivo*.

**Table 1:** Main strategies to facilitate endosomal escape of nucleic acid nanoformulations.

Strategy	Mechanism	Selected references
**Co-delivery with lysosomotropic agent**	Buffering luminal pH, osmotic swelling of endosomal vesicles	Mesoporous silica nanoparticles conjugated with PEG and PDEAEMA for delivery of DNA or siRNA. Loading of these particles with chloroquine significantly improved efficacy of gene delivery or gene silencing in B16F10 melanoma cells (Bhattarai et al., 2010)
**Co-delivery with photosensitizers**	Light-induced generation of reactive oxygen species and lipid oxidation result in enhanced permeability of endosomal membrane	Micelles based on triblock copolymer/DNA/dendrimeric photosensitizer caused 100-fold photoenhanced gene expression in HeLa cells and site-specific transfection of tumors (Nomoto et al., 2014)
**Chemical functionalization with CPPs:** • **TAT peptide** • **Pore-forming peptides** • **Fusion peptides**	• For TAT peptide the mechanism is unclear • Pore-forming and fusion peptides upon endosomal acidification undergo transition from random coil (pH 7) to α-helical (pH 5.5) conformation that increases hydrophobic interaction of peptide and membrane bilayer	• Intratumorally injected TAT-modified DNA-containing liposomes much more effectively transfected Lewis lung carcinoma tumors than unmodified counterparts (Torchilin et al., 2003) • Polyplexes conjugated with melittin caused effective gene silencing in H1299 lung carcinoma cells and in lung tissue upon intratracheal administration (Feldmann et al., 2018)
**Incorporation of polyamines**	Osmotic swelling and electrostatic interaction with endosomal membrane	Cyclodextrin and PEI functionalized mesoporous silica nanoparticles efficiently accumulated in MDA-MB-231 tumors and caused gene silencing (Shen et al., 2014)
**Incorporation of amphiphatic polycations**	Perturbation of endosomal membrane after pH-triggered dissociation of shielding moieties	DPCs comprising PBAVE conjugated via pH-triggered linkers with siRNA, PEG and GalNAc were able to effective delivery of genetic cargo to hepatocytes *in vitro* and *in vivo* and cause significant gene silencing (Rozema et al., 2007)

The second approach facilitating non-viral transfection is treatment of cells with photosensitizers, also known as “photochemical internalization” (Oliveira et al., 2007; Zamora et al., 2014). Upon uptake, photosensitizers cause light-induced oxidative disruption of endosomal membranes resulting in enhanced gene delivery (Selbo et al., 2010). It should be noted that photosensitizers are widely used for photodynamic therapy and considered as safe agents for systemic administration. However, for better efficacy, the photosensitizer should be incorporated into the structure of the gene delivery nanocarrier. The advantage of this approach has been demonstrated *in vivo* for nanoparticles comprising of plasmid DNA as the genetic payload, PEG-poly{*N*-[*N*-(2-aminoethyl)-2-aminoethyl]aspartamide}-poly(L-lysine) for DNA complexation and providing long circulation, and dendrimeric phthalocyanine as the photosensitizer. Obtained micelles demonstrated 100-fold photoenhanced gene expression in HeLa cells and site-specific transfection of tumor cells (Nomoto et al., 2014).

Another method for improvement of endosomal escape of non-viral vectors is modification of their surface with cell-penetrating peptides (CPPs). Numerous peptidic moieties have been proposed for this aim including fusogenic virus-derived (Wagner et al., 1992) or synthetic peptides such as GALA (Simões et al., 1999) and KALA (Min et al., 2006), or pore-forming viral- (Zauner et al., 1995) and bacterial-derived (Saito et al., 2003; Lorenzi and Lee, 2005) peptides. Moreover, peptides from insect venoms such as melittin have been extensively studied for optimization of lipoplex- and polyplex-mediated transfection of fibroblasts (Legendre et al., 1997), H1299 lung carcinoma cells and lung tissue (Feldmann et al., 2018). All these peptides have a similar mechanism of membranotropic activity, mediated by a change of endosomal pH. Acidification of the endosomal lumen leads to conformational transition of these peptides from a random coil to an amphipathic α-helix, which interacts with membrane phospholipids and causes pore formation or fusion of endosomal membrane with, for example, the viral envelope (Varkouhi et al., 2011). Another example of CPP is TAT peptide derived from the transcriptional activator protein of human immunodeficiency virus type 1 (HIV-1). Although the mechanisms of membrane disruption caused by TAT peptide are unclear, it was successfully exploited *in vivo* to improve local polyplex-mediated gene delivery to lung tissue (Kleemann et al., 2005) and Lewis lung carcinoma tumors (Torchilin et al., 2003). In spite of improvement in transfection efficacy, clinical translation of delivery systems comprising peptide moieties holds potential immunogenicity concerns.

As mentioned previously, there are some inorganic nanocarriers for nucleic acid delivery which are unable to induce endosomal escape. Their modification with polyamines, such as poly(amidoamine)s or PEI, facilitate endosomal release and significantly enhances transfection efficacy both *in vitro* and *in vivo* (Shen et al., 2014; Ngamcherdtrakul et al., 2015; Luo et al., 2017).

Finally, incorporation of amphiphatic polycations (Wakefield et al., 2005) into carriers can also improve endosomal release of nucleic acids. As an example of this approach, dynamic polyconjugates (DPCs) developed for siRNA delivery to hepatocytes contain membrane-active poly(vinyl ether) polymer, termed PBAVE. This polymer is shielded by PEG for prolonged circulation and *N*-acetylgalactosamine (GalNAc) moieties for targeting hepatocytes (Rozema et al., 2007). Positive pre-clinical results (99% silencing of liver genes in non-human primates) made it possible to begin clinical trials of this approach (Yin et al., 2014). Harnessing of this technology for tumor-targeted siRNA delivery holds a great promise for anti-cancer gene therapy.

## Nuclear Entry of DNA

Transcription of foreign DNA requires its nuclear-targeted delivery. However, only relatively short DNA fragments (less than 200–300 bp) are able to penetrate the nucleus by passive diffusion through the NPC (Ludtke et al., 1999). As far as the approximate length of therapeutic DNA reaches some kbp, its delivery to the nucleus via NPC is highly challenging and unlikely. Some attempts to improve NPC-mediated DNA delivery have been made (**Table 2**). The first approach considered covalent conjugation of DNA with a peptidic nuclear localization signal (NLS), responsible for active importin-mediated nuclear transfer of large cytoplasmic proteins. This approach improved nuclear transfer of plasmid DNA with attached SV40 viral NLS peptide in digitonin-permeabilized cells, but not in intact cells after microinjection of the modified DNA (Sebestyén et al., 1998). The similar approach in relation to linear 3.3 kbp DNA largely enhanced its nuclear uptake (Zanta et al., 1999), but a later study indicated that there is no benefit of NLS conjugation to DNA for improved gene delivery with non-viral carriers (van der Aa et al., 2005). Another approach considered inclusion of nucleotide sequences into DNA which can be recognized by cytoplasmic transcription factors containing NLS, and then transported into the nucleus (Dean et al., 1999; Mesika et al., 2001). However, nuclear translocation of cytoplasmic transcription factors requires activation of certain signaling pathways in cancer cells that strongly limits application of this approach. Furthermore, this technology has not been validated *in vivo*. Next, the use of *trans-*cyclohexane1,2-diol (TCHD) was proposed to improve NPC-mediated nuclear uptake of DNA. This amphipathic alcohol temporary perturbed the barrier function of the NPCs and facilitated nuclear entry of dextrans and naked plasmid DNA. Moreover, TCHD treatment of cells enhanced lipoplex-mediated transfection (Vandenbroucke et al., 2007). However, application of this strategy is also limited by toxicology aspects of TCHD use *in vivo*. It should be noted that the barrier function of NPC can be decreased by conjugation of gene delivery nanocarriers with a ligand moiety to the cytoplasmic glucocorticoid receptor. Interaction with this receptor induces NPC dilation up to 300 nm in diameter and nuclear translocation of ligand/receptor complex (Yao et al., 2013). The advantage of this strategy was shown for polyplexes based on hyaluronic acid (HA)-PEI-dexamethasone block-copolymer. These nanoparticles displayed higher efficacy of gene delivery to cancer cells and tumors compared with non-modified counterparts (Fan et al., 2013). Targeting a nanocarrier with all-*trans*-retinoic acid (ATRA) to cytoplasmic retinoic acid receptor also promotes nuclear translocation of ligand/receptor complex via a similar mechanism as in the case of glucocorticoid receptor. In spite of promising *in vitro* results for ATRA-targeted polyplex-based carriers (Park et al., 2009), *in vivo* translation of this strategy for cationic liposomes did not lead to enhanced efficacy of gene delivery as compared with non-targeted counterparts (Charoensit et al., 2010). However, they demonstrated improved anti-tumor effect presumably due to ATRA-mediated TNF-α-induced apoptosis in cancer cells (Charoensit et al., 2010).

**Table 2:** Main strategies to facilitate nuclear uptake of DNA delivered using non-viral vectors.

Strategy	Proposed mechanism	Outcome
**Conjugation of NLS to DNA**	Importin-mediated transfer of DNA to the nucleus via NPC	Very contradictory results, the latest study indicated no transfection augmentation after NLS conjugation (van der Aa et al., 2005); no *in vivo* follow-up
**Modification of nucleotide sequence with transcription factor binding sites**	Interaction of DNA with transcription factors in cytosol followed by importin-mediated translocation to the nucleus	Improved transfection efficacy of cell culture (Dean et al., 1999; Mesika et al., 2001); no *in vivo* follow-up
**Pre-treatment with TCHD**	Perturbation of NPC barrier function for macromolecules	• Enhanced lipoplex-mediated transfection (Vandenbroucke et al., 2007); no *in vivo* follow-up
**Conjugation of nanocarrier with ATRA**	Interaction with retinoic acid receptor promotes NPC dilation and nuclear translocation of ligand/receptor complex	Enhanced *in vitro* transfection mediated by ATRA-modified polyplexes (Park et al., 2009) • No augmentation of transfection efficacy in A549 tumor nodules in lungs upon intravenous administration of ATRA-modified cationic liposomes (Charoensit et al., 2010)
**Conjugation of nanocarrier with dexamethasone**	Interaction with glucocorticoid receptor promotes NPC dilation and nuclear translocation of ligand/receptor complex	HA/PEI-dexamethasone/DNA ternary complexes demonstrated improved transfection of cancer cells and more efficiently inhibited HepG2 tumor growth (Fan et al., 2013)
**Optimization of DNA uptake by daughter cell nuclei during mitosis**	• Pre-condensation of DNA with a CdK1-responsive peptide before lipoplex formation was thought to promote DNA protection in G_0_/G_1_, S, and G_2_, but dissociation and nuclear uptake during mitosis • Pre-condensation of DNA with chromatin targeting peptides before lipoplex formation was thought to provide binding of DNA with chromosomes during mitosis and improved nuclear uptake	Both approaches slightly increased transfection efficacy of HeLa and A549 cultured cells by lipoplexes with pre-condensed DNA, but irrelevant to the nature of the peptide used for DNA pre-condensation (Remaut et al., 2014); no *in vivo* follow-up

It is commonly accepted that the primary way of foreign DNA entry is a passive entrapment during mitosis, when the nuclear envelope is disassembled starting from late prophase up to late anaphase, and its components (proteins and membranes) are associated with the endoplasmic reticulum (Symens et al., 2012). Thus, the efficiency of foreign DNA delivery in dividing cells will depend on the amount of intact DNA present at the moment of cell division nearby the chromatin. Live imaging experiments carried out for lipoplexes (Kirchenbuechler et al., 2016) and polyplexes (Durymanov et al., 2015) has shown that 85–90% of transfected cells acquired the gene expression definitely after passing through mitosis. In light of the significant contribution of cell division to transfection, two strategies have been proposed to optimize DNA engulfment into daughter cell nuclei. The first approach proposed pre-condensation of DNA with a CdK1-responsive peptide before formation of lipoplexes. It was hypothesized that after the endosomal escape, these peptides stay in complex with DNA in cytosol and protect DNA from cytosolic nucleases, but dissociate during mitosis providing nuclear entrapment of the higher amount of non-damaged DNA. It turned out that DNA pre-condensation slightly increased transfection efficacy, but irrelevant to the nature of the peptide used for DNA pre-condensation (Remaut et al., 2014). The second strategy, proposed in the same study, aimed at specific anchoring of plasmid DNA to chromatin in newly formed nuclei due to pre-condensation of DNA with chromatin targeting peptides from the Kaposi’s sarcoma-associated herpes virus. However, in spite of increased transfection, the nature of the peptide also did not seem to be the main reason for improvement (Remaut et al., 2014).

Mitosis-dependent transfection of cells may be significantly limited in cell lines with infrequent cell division. Remarkably, even without NLS and other nucleus-targeting sequences, lipoplex- or polyplex-formulated DNA can reach the nucleus in a mitosis-independent manner according to live-cell microscopy data (Durymanov et al., 2015; Kirchenbuechler et al., 2016). Some data supporting this finding were previously obtained by additional indirect methods including flow cytometry (Brunner et al., 2002; Grosse et al., 2006; Matz et al., 2013). The mechanisms underlying mitosis-independent non-viral gene delivery are still unknown. In the case of polyplexes, they are mostly attributed to the ability of polycations to permeabilize the nuclear envelope (Grandinetti and Reineke, 2012; Grandinetti et al., 2012), whereas mitosis-independent nuclear entry of lipoplex-formulated DNA is thought to occur via direct fusion of lipoplex-containing endosomes with the nuclear membrane (Elouahabi and Ruysschaert, 2005). However, direct experimental proofs of the mentioned mechanisms are still lacking.

## Impact of Intracellular Barriers on Non-Viral Transfection

Intracellular penetration of nucleic acid nanoformulations seems to be a relatively effective process, although several studies state that cellular uptake efficacy has a direct impact on the transfection process (Sakaguchi et al., 2008; Trusov et al., 2011; Ulasov et al., 2011; Bishop et al., 2016). Hence, modulation of the cellular internalization process via variation of nanoparticle design and receptor targeting may accelerate uptake and increase internalized genetic material amount, resulting in higher transfection efficacy.

Endosomal escape, or the endosomal membrane, is a significant barrier which strongly affects the success of nucleic acid non-viral delivery. Time-lapse microscopy studies determined that there is from one to five release events per cell over several hours in the case of both lipoplexes (Wittrup et al., 2015) and polyplexes (ur Rehman et al., 2013). In the latter study the authors also estimated the frequency of release events by transfection of cells with nanoparticles carrying a mixture of two differently labeled short (∼20 bp) DNA fragments in a 1:1 ratio (red and green), or a mixture of two types of nanoparticles, each containing only single-labeled DNA (either red or green). In case of lipo- or polyplexes with double-labeled genetic payload all transfected cells contained both types of DNA fragments released from endosomes, whereas in the case of the mixture of nanoparticles with single-labeled DNA the values were different. For polyplexes, two types of DNA fragments were present in 23% of transfected cells whereas the other 77% of transfected cells contained either green or red fragments. For lipoplexes, 80% of transfected cells contained two types of oligonucleotides and 20% were single-positive. These values enable us to calculate an average number of release events per cell for lipoplexes N_lp_ and polyplexes N_pp_. The probabilities that transfected cells will get two types of DNA fragments in assumption of their release from different endosomal vesicles are 1 – 0.5*^Nlp-1^* = 0.8 for lipoplexes, and 1 – 0.5*^Npp-1^*= 0.23 for polyplexes (where *N_lp_* ≥ 1 and *N_pp_* ≥ 1, because we consider only transfected cells). Resulting in values of *N_pp_* = 1.4 and *N_lp_ =* 3.3, indicating almost twofold higher efficiency of endosomal escape in case of lipoplexes, although this conclusion only applies to certain carriers (22 kDa linear PEI for polyplexes and Lipofectamine 2000 for lipoplexes) used in this study. Actually, between one and five release events per cell over some hours have been observed for polyplexes (ur Rehman et al., 2013) and lipoplexes (Wittrup et al., 2015), indicating that only a very few number of nanoparticles overcome the endosomal barrier and contribute to transfection.

Regarding crossing the nuclear membrane barrier, numerous studies indicate a strong dependence of cell transfection on mitosis where the nuclear envelop is temporarily dismantled. Real-time microscopy of polyplex-mediated transfection has shown that cell division can result in only one post-mitotic transfected cell of the two; indicating non-uniform partitioning of plasmid DNA between the two daughter cells (Durymanov et al., 2015). Therefore transfection of the dividing cell is likely determined by only a few intact plasmids (probably originating from a single polyplex particle) in cytosol in close proximity to chromatin at the moment of mitosis. Most probably, the average number of intact DNA molecules released from Lipofectamine 2000-based lipoplexes is higher because singly transfected daughter cells were not detected during microscopy tracking of cells (Kirchenbuechler et al., 2016). It should be noted that transfection probability of actively dividing cells also depends on the cell cycle phase when DNA was released from endosome. For example, the highest transfection efficacy was observed for cells which entered mitosis 5–13 h after polyplex addition; approximately corresponding to early S phase (Durymanov et al., 2015). However, this time seems to be very variable for different cell lines and non-viral carriers. Interestingly, both lipo- and polyplexes can transfect non-dividing cells in culture. Because the vast majority of cancer cells in clinical tumors divide much slower than in the investigated cultured cell lines, the mechanism of mitosis-mediated DNA translocation might not be clinically relevant. In fact, transfection in non-dividing cells can occur, but efficacy of this process is too low and the mechanisms of nuclear DNA uptake are not determined. Elucidation of such mechanisms will likely provide effective tools for transfection of quiescent cells.

## Optimization of Nucleic Acid Nanoformulations for *In Vivo* Translation

Despite the importance of surmounting intracellular barriers by non-viral nanoparticles, the most significant fraction of effort to date is focused on safety/toxicity issues, improving biodistribution and tumor selectivity, and overcoming extracellular barriers, particularly, the tumor stromal barrier (Wang et al., 2015; Oliveira et al., 2016; Rezaee et al., 2016), because intratumoral extravasation and penetration of nucleic acid nanoformulations are very limited (Durymanov et al., 2013, 2016; Shen et al., 2014). These efforts are critical for the *in vivo* translation of the nucleic acid nanoformulations.

It should be noted that some aspects of nanoparticle tuning for *in vivo* translation are contradictory to the transfection efficacy on a cellular level. These discrepancies originate from the desired nature and properties of the nanocarrier. First, it must have a positive charge; either functional groups for electrostatic complexation or covalent attachment of nucleic acids. The positive charge of the nanocarrier protects genetic cargo form extracellular nucleases, facilitates interaction with the cell surface and uptake, and provides endosomal escape by interaction with anionic phospholipids in endosomal membrane. However, positive surface charge may also cause fast elimination of nucleic acid nanoformulations from circulation and off-target delivery of nucleic acids. The most common strategy to improve biodistribution in intravenously administered nanotherapeutics is shielding with neutral hydrophilic polymers like polyethylene glycol (PEG). PEGylation not only extends circulation time, but also improves diffusion-mediated permeation of tumor stroma due to minimization of electrostatic interactions with extracellular matrix (Nance et al., 2014). At the same time, functionalization with PEG impairs cellular uptake and may reduce endosomal escape efficacy. To solve this “PEG dilemma,” different stimuli-responsive strategies can be exploited enabling, for example, a shedding PEG coat in the tumor environment or endosomes resulting in more efficient cellular uptake, endosomal release and gene silencing (Salzano et al., 2015). Another promising stimuli-responsive delivery system for nucleic acid delivery is lipid nanoparticles (LNP) based on lipids with protonable headgroups at endosomal pH (Del Pozo-Rodríguez et al., 2016). In extracellular space these carriers have neutral charge that provide long circulation times and higher tumor uptake, while in endosomes of cancer cells they acquire positive charge facilitating release of genetic cargo. Thus, the mentioned advantageous properties of LNPs resulted in their early clinical translation (Zatsepin et al., 2016), although the efficacy of endosomal escape for such LNPs (Gilleron et al., 2013), as well as cellular uptake (Sahay et al., 2013), are not very high and require additional optimization.

Another requirement of nanocarriers for delivery of nucleic acids is efficient internalization, which besides positive surface charge, can be modulated by functionalization with a ligand moiety. This modification for improvement of non-viral vectors on a cellular level has minimal conflict with *in vivo* application enhancing selectivity of tumor-targeted delivery, but potentially may impair nanoparticle penetration of tumor stroma due to “binding site barrier” effect detected for small targeted nanoparticles (Lee et al., 2010). For solving this problem, modification of the nanocarrier with tumor-penetrating peptides can exploited. This technology enables tumor-specific extravasation and delivery of nucleic acid and other nanoformulations deep into the tumor parenchyma (Ren et al., 2012).

Finally, as opposed to *in vitro* use, very strict analysis of safety and biodegradability properties of nanocarriers should be carried out for clinical translation. Many types of cationic lipids or polycations commonly used for *in vitro* applications cannot be translated *in vivo* due to these issues. Additionally, modification of nanocarriers with virus- or bacteria-derived CPPs potentially holds immunogenicity limitations.

As a result, in spite of a diversity of nucleic acid delivery systems, only a limited number fulfills these *in vivo* requirements and maintains relatively high efficacy of overcoming intracellular barriers. We believe that ongoing (Yin et al., 2014; Zatsepin et al., 2016) and future clinical trials of these carriers including GalNAc conjugates, LNPs, and DPCs will provide an effective tool for anti-cancer gene-based therapy and clarify future prospects.

## Conclusion

Despite progress in studying mechanisms of cell transfection by non-viral vectors and elucidation of the impact of intracellular barriers on transfection efficacy, some important information is still lacking. For instance, it is still unknown how endosomal phospholipids interact with nucleic acid nanoformulations. Based on knowledge about endosome maturation process, we hypothesized that negatively charged BMP/LBPA might be an important participant of this process. Additionally, clinically relevant mitosis-independent mechanisms of DNA translocation into the nuclei of cancer cells are unknown, ineffective, and not well-managed. Probably, their identification and exploitation will significantly improve transfection efficacy of quiescent tumor cells with DNA-containing non-viral nanoformulations.

Elucidation of transfection mechanisms is very important as it creates a strong basis for novel directions of nanocarrier improvement. We believe further analysis/understanding of endosomal escape and nuclear localization mechanisms will advance nucleic acid nanoformulations toward higher efficiency and enable clinical translation for numerous applications including cancer gene-based therapy.

## Author Contributions

Both authors listed have made a substantial, direct and intellectual contribution to the work, and approved it for publication.

## Conflict of Interest Statement

The authors declare that the research was conducted in the absence of any commercial or financial relationships that could be construed as a potential conflict of interest.
